# Case Report of a 13-Year-Old Female With Trauma Secondary to a Fall From a Golf Cart, Found to Have Intraparenchymal & Subarachnoid Hemorrhages With Transverse Sinus Thrombosis

**DOI:** 10.7759/cureus.27251

**Published:** 2022-07-25

**Authors:** Umberto M Donato, Sebastian Donato, Andrew Galligan

**Affiliations:** 1 Pediatric Oncology, Moffitt Cancer Center, Tampa, USA; 2 Pediatric Oncology, Tampa General Hospital, Tampa, USA; 3 Radiology, Moffitt Cancer Center, Tampa, USA; 4 Pediatric Hematology Oncology, University of South Florida Morsani College of Medicine, Tampa, USA; 5 Pediatric Oncology, The Ohio State University, Columbus, USA

**Keywords:** mr venography, mrv, pediatric head trauma, pediatrics emergency, anticoagulation therapy, cerebral venous sinus thrombosis (cvst)

## Abstract

Cerebral venous sinus thrombosis (CVST) is the occlusion of cerebral veins of the brain secondary to blood clot formation. These can result in increased intracranial pressure, cerebral edema, and may even have fatal consequences such as a stroke. Despite CVSTs being considered a rare pathology, these are said to have an increased incidence in the pediatric population. These individuals with CVST are often asymptomatic causing physicians to often overlook and delay possibly life-saving interventions. The current literature is lacking on CVST examples in the “older” range of the pediatric population, specifically teenagers. Here we present the case of a 13-year-old female with trauma secondary to a fall from a golf cart, who was found to have intraparenchymal and subarachnoid hemorrhages with transverse sinus thrombosis.

## Introduction

A cerebral venous thrombosis (CVST) is defined as the thrombosis of deep cerebral veins, dural venous sinuses, cortical veins, and/or cerebral venous sinus. In the past CVSTs were only thought to occur in severe head injuries, however, data has proven that CVSTs can occur in closed head injuries as well [1]. Moreover, CVSTs are a quite rare yet clinically relevant pathology with an incidence of less than 1/100,000 in children under 18 years of age [2]. The presentations can range from a mild headache to violent seizures and even death [3]. Be that as it may, when CVSTs are identified promptly in their course, patients can then undergo adequate treatment and oftentimes achieve full recovery [4]. The current standard techniques used to provide a diagnosis of CVSTs includes magnetic resonance imaging venography (MRIV) and computed tomography venography [5]. In cases of acute posttraumatic CVST, it is of increasing importance to have magnetic resonance venography (MRV) technology at hand in order to expedite the anticoagulant therapy interventions for thrombosis. Altogether, we present the case of an otherwise healthy 13-year-old female who sustained a transverse sinus thrombosis and a subarachnoid bleed as a consequence of trauma secondary to a fall from a moving vehicle.

## Case presentation

A 13-year-old female presented as a trauma alert following a fall from a golf cart. The golf cart was traveling around 15 mph (the patient was sitting at the back) when she fell off and landed on the back of her head. Bystanders reported the patient lost consciousness for approximately one minute before being transported via ambulance to the ED. The patient had a Glasgow coma scale (GCS) score of 15 (normal) on initial evaluation by the emergency medical services at the scene but bystanders mentioned the first few minutes before the ambulance arrived the patient appeared “dazed and confused”. En route to the ED, she developed nausea and vomiting for which Zofran 4 mg and 250 ml normal saline (NS) bolus were given. The patient was placed on a C-collar prophylactically. At the ED she had another episode of emesis and developed a slight nose-bleed which resolved spontaneously an hour later. A review of systems was unremarkable except for a posterior scalp hematoma and left-sided ear pain (throbbing) which the patient labeled a 6/10.

**Figure 1:**
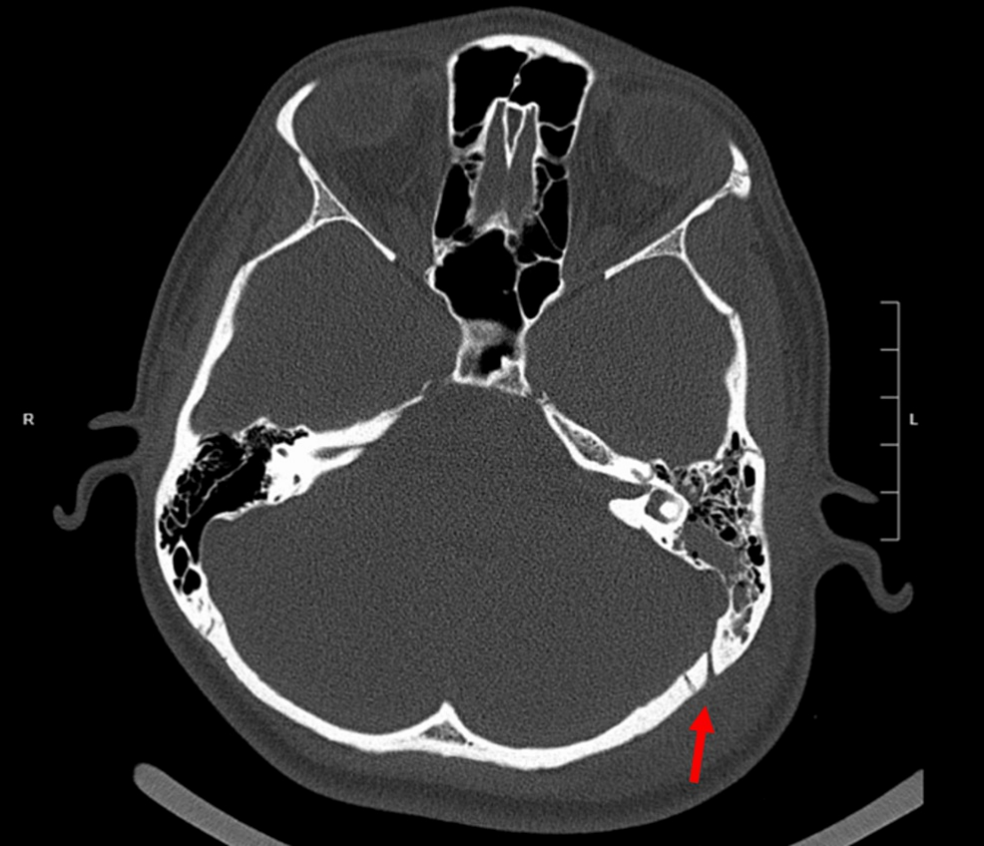
Head CT Primary CT Impression: Left basilar skull fracture communicating with the ipsilateral mastoid air cells.

Labs including a complete blood count (CBC), comprehensive metabolic panel (CMP), and imaging (head & C-spine CT/chest X-ray) were ordered soon thereafter. The initial differential before imaging and lab data included intraparenchymal hemorrhage (IPH), concussion, brain contusions, and/or skull fractures. Vital signs were within reference values for the patient’s age. The C-spine CT and chest X-ray were both unremarkable for any acute traumatic abnormalities. The head CT, however, was notable for intracranial hemorrhage with contrecoup injury along the frontal lobe and left temporal lobe. There was also a left basilar skull fracture communicating with the ipsilateral mastoid air cells with possible left dural venous sinus involvement (Figure 1). Overall, the main impressions of the head CT were: bilateral frontal IPH, small (left temporal) subarachnoid hemorrhage, occipital subgaleal hematoma, left posterior temporal bone fracture, and a possible left dural venous sinus injury. The results of the lab tests (Table 1 and Table 2) were mostly unremarkable except for the decreased platelet count and partial thromboplastin time; such lab values are seen in patients with cerebral venous sinus thrombosis (CVST) [6].

**Table 1:** Complete blood count (CBC) values Comment: Therapeutic reference range for unfractionated heparin six hours post bolus 57.0-97.0 seconds (equivalent to 0.30 - 0.7 IU/mL heparin anti-Xa)

Table 1: CBC Values			
	Values at presentation	Values at discharge	Reference values
White blood cell (10*3/uL)	15.63	6.4	4.6-10.2
Red blood cell (10*6/uL)	4.26	3.61	4.04-5.48
Hemoglobin (g/dL)	12.3	10.6	12.2-16.2
Hematocrit (%)	39.5	32.3	38.0-47.9
Mean corpuscular volume (fL)	92.7	89.5	80-97
Mean corpuscular hemoglobin (pg)	26.7	29.4	27-31.2
Mean corpuscular hemoglobin concentration (g/dL)	28.9	32.8	27.0-31.2
Platelet count (10*3/uL)	133	168	142.0-424.0
Mean platelet volume (fL)	11.0	11.5	9.4-12.4

**Table 2:** Partial thromboplastin time lab values of our patient

Table 2: PTT Values			
	Value at presentation	Value at discharge	Reference Values
Partial thromboplastin time (PTT) in seconds	16.6	27.5	24.0-36.5

**Figure 2:**
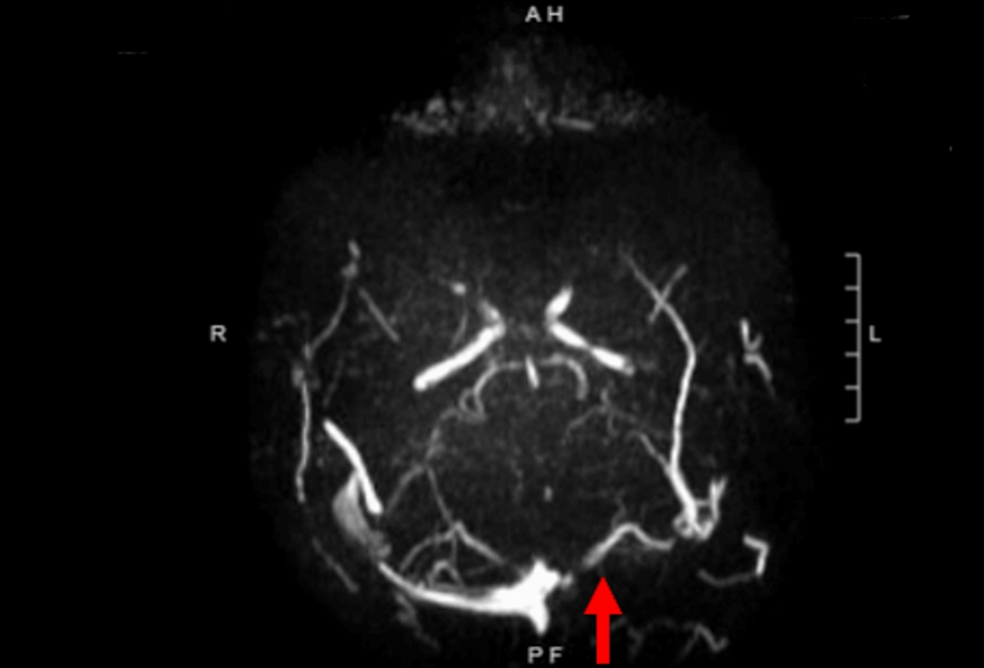
Axial head magnetic resonance venography (MRV) Empty delta sign suspicious for thrombosis in the left transverse sinus

**Figure 3:**
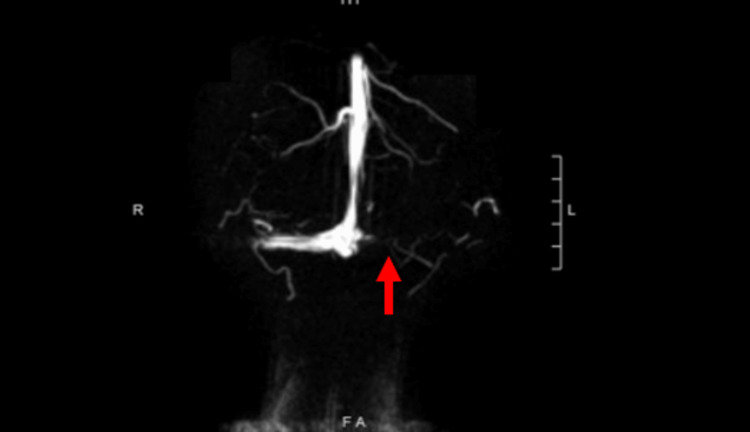
Coronal head magnetic resonance venography (MRV) Empty delta sign suspicious for thrombosis in the left transverse sinus

Due to the CVST suspicion and the history of significant head trauma, magnetic resonance venogram (MRV) and non-contrast head MRI were also ordered. The MRV evidenced a no-flow signal in the left transverse sinus and a limited flow signal in the distal right transverse sinus, aligning with the initial CVST suspicion (Figure 2 and Figure 3). Due to this, it was not possible to exclude a dural venous sinus thrombosis diagnosis. Moreover, the head MRI revealed evolving bifrontal and left temporal hemorrhagic contusions with similar supratentorial subarachnoid blood products and moderate left mastoid air cell effusion. After these results, the patient was then admitted to the pediatric intensive care (PICU) unit for two midnights and was started on 500 mg of Keppra daily as a prophylactic dose for traumatic brain injury-related seizures. The patient underwent quarterly one-hour checks in order to assure she remained stable. The c-collar on the patient was removed and she was started on an anticoagulation heparin drip for a therapeutic goal of 0.3 anti-Xa. The plan was to repeat MRI/MRV of the brain once the anti-Xa therapeutic goal was achieved. Once the expected anti-Xa values were reached, the second MRI/MRV was performed and the CVST was no longer visible. The patient was discharged two midnights after PICU admission.

## Discussion

Undoubtedly, CVST is a fairly common pathology seen in the clinical setting [7]. Be that as it may, the incidence of this condition is nearly double in the pediatric population [7]. Noting the elevated incidence levels of CVST, specifically in the pediatric population, physicians must be well informed and understand that this particular disorder is potentially fatal and can be easily overlooked. In severe cases of CVST, mortality has been cited to be up to 34% [8]. Most patients with CVSTs present with pathognomonic symptoms of elevated intracranial pressure including but not limited to headaches. Headaches are considered the most common symptom and can often present by themselves in the absence of an intracerebral lesion [9].

That said, CVST is characterized by an ample array of onset types and symptoms thus making it increasingly difficult to diagnose. Due to the often non-specific presentations of this pathology, CVST diagnoses are centered on clinical suspicion due to the patient's symptoms and contrast imaging (CT, MRI, MRV). As of now, the available literature is currently lacking data pertinent to the clinical presentation of older pediatric patients (teens) with CVSTs. As mentioned earlier it is very likely that such diagnoses are often missed and/or delayed in this particular population. For example, toddlers are more frequently diagnosed with CVSTS since these individuals usually present with focal neurological changes and headaches whereas these types of presentations are said to be more common in older pediatric groups [10-11]. As a physician, it is important to be aware of these changes to avoid overlooking possible CVST diagnoses. That said, CVST after head trauma is fairly uncommon. However, when fractures of the temporal bone are seen, patients are 50% likely to suffer from CVST. In the case of our patient, she was diagnosed with a left temporal bone fracture, left temporal subarachnoid hemorrhage (SAH), and finally a left transverse sinus CVST. Due to the patient's presentation with a headache, vomiting, nausea, and history of a temporal bone fracture, the possibility of CVST was always considered.

Retrospectively, however, the SAH seen in our patient proved to be quite puzzling. Due to the concomitant location of the CVST, the bone fracture, and the SAH, it was hard to define whether the SAH was secondary to the CVST or secondary to the initial head trauma that brought the patient to the ED. The CVST could have led to hypertension of the cerebral venous system and caused the rupture of dilated bridging subarachnoid cortical veins [12], or it could have just been a direct result of the trauma. It is our opinion that the SAH in our patient was most likely a direct result of the trauma since only 3% of SAHs are said to be a result of a concomitant CVST [13]. Nevertheless, due to our patient's symptomatic presentation and history of head trauma head MRIs and MRVs were performed immediately. The MRV imaging results illustrated the “empty delta sign” [14]. This sign represented in our case, contrast enhancement flowing around the comparatively hypodense region of the thrombosed transverse cerebral sinus. This radiological finding along with the decreased PTT and platelet count lab values that were highly suspicious for a venous thromboembolic event helped us provide a definitive CVST diagnosis [6].

After the CVST diagnosis, the patient was started on a heparin-based anticoagulant therapy since it is considered the first line of treatment for patients diagnosed with a CVST [14]. After two days of the anticoagulant therapy, the patient’s CVST resolved, and was discharged. Overall, this case served to highlight the importance of imaging and lab values in providing a CVST diagnosis in a rare pediatric case with concomitant SAH and a temporal bone fracture. 

## Conclusions

Despite the fact that traumatic head injuries are a rare cause of CVSTs, this case serves to underscore the often overlooked incidence of this pathology in the pediatric population. Moreover, this case illustrates the importance of considering CVST diagnoses in patients presenting with SAH and headaches without evidence of a brain aneurysm. Furthermore, the present CVST example may serve to underscore the efficacy of gold standards of diagnosis and treatment of this pathology. 
